# Neuroblastoma cell growth and invasiveness is modulated by the activity of N-acetylglucosaminyltransferase-III

**DOI:** 10.1371/journal.pone.0350822

**Published:** 2026-06-08

**Authors:** Adam P. Burch, M. Kristen Hall, Ruth A. Schwalbe

**Affiliations:** Department of Biochemistry and Molecular Biology, Brody School of Medicine, East Carolina University Greenville, Greenville, North Carolina, United States of America; University of Mississippi, UNITED STATES OF AMERICA

## Abstract

The presence of complex N-glycans with β1,6-N-acetylglucosamine (GlcNAc) on cells has long been associated with cancer. For this reason, the role of N-acetylglucosaminyltransferase-III (GnT-III, *MGAT3*) to hinder terminal N-glycan processing by the addition of β1,4-GlcNAc residues (bisected N-glycan), has earned GnT-III a reputation as a tumor suppressor. In this study, by the creation and characterization of human *MGAT3* knockout in the Be(2)-C cell line, a NB cell line, (*MGAT3-/-*), we solidify GnT-III activity as a suppressor of NB progression by showing that the loss of GnT-III coincided with increased cell proliferation and cell spheroid invasion, along with cell migration, and cell-cell adhesion, and modified cell types. Lectin blotting results indicated that bisected N-glycans were greatly diminished in *MGAT3-/-*. Further GNL and L-PHA binding suggested that oligomannose and β1,6-GlcNAc branched N-glycans were raised in *MGAT3*-/- relative to the BE(2)-C cell line. Cellular phenotypes of *MGAT3-/-* could be rescued by transient expression of *Mgat3* cDNA. Likewise, altered levels of bisecting GlcNAc N-glycans in a rat NB cell line (NB_1) overexpressing GnT-III had reduced cell-cell adhesion, cell migration, and cell invasiveness. We conclude that lowered GnT-III activity promotes cell invasiveness and growth in NB, and speculate that oligomannose and β1,6-GlcNAc branched N-glycans are key determinants.

## Introduction

Marked by poor clinical outcome and a high level of heterogeneity, neuroblastoma (NB) is a devastating pediatric cancer responsible for an estimated 15% of pediatric cancer related deaths [1]. Despite the application of aggressive multimodal therapies, the 5-year survival rate remains under 50% for high-risk patients, highlighting a need for new and improved diagnostic/therapeutic targets [2,3]. One such target could be the process of N-glycosylation [4]. In NB, the expression of glycosyltransferases, enzymes involved in N-glycan processing, have been shown to serve as predictors of patient outcome [5–7]. In addition, modifying the number and elongation of branches on N-glycans via decreased and increased expression of glycosyltransferases (e.g., *B4GALNT3*, *MGAT1* and *MGAT2* genes) of NB cells has been shown to be a powerful method of altering cellular properties such as proliferation, invasion, and cell-cell adhesion [6,8–12].

N-glycosylation is a template-free, co-/post-translational process that occurs upon the entry of a protein into the endoplasmic reticulum (ER). There are three major types of N-glycans: oligomannose, hybrid, and complex [13]. While N-glycans are synthesized and attached to nascent peptide chains in the ER, the bulk of N-glycan processing occurs post-translationally within the Golgi apparatus. Terminal N-glycan processing within the Golgi includes the conversion of oligomannose N-glycans to hybrid N-glycans via N-acetylglucosaminyltransferase-I (GnT-I), and subsequent conversion of hybrid to complex types of N-glycans via GnT-II [13]. Moreover, hybrid and complex N-glycans can give rise to an impressive display of structural diversity, driven by the actions of other Golgi resident glycosyltransferases: fucosyltransferases (FUTs), sialyltransferases (STs), galactosyltransferases (GALTs), and other GnTs [14–17]. Of importance to this study, is GnT-III, which can add a β1,4-N-acetylglucosamine (GlcNAc) to the central mannose of the conserved pentasaccharide of hybrid or complex N-glycans. These types of N-glycans are referred to as bisected N-glycans. The insertion of the bisecting GlcNAc residue effectively halts further N-glycan processing [18]. For this reason, GnT-III activity is thought to serve an antagonistic role to other medial- and trans-Golgi glycosyltransferases processing, such as additions by GnT-IV/V, FUTs GALTs, and STs [18–20].

GnT-III has often been considered a tumor suppressor, primarily for its antagonistic relationship with GnT-V (*MGAT5*), a tumor promoter, in several cancers [19,21,22]. In a human NB cell line, cell migration was increased corresponding to a decrease in GnT-III and a subsequent increase in GnT-V activity [19]. Moreover, reduced GnT-III activity increased NB cell proliferation and cell-cell adhesion [23]. In contrast, GnT-III levels appeared to slightly decrease with NB cell differentiation [24]. Further NB with favorable prognosis have been shown to express high levels of *MGAT5* transcripts [7] which correlated with studies showing that high levels of oligomannose N-glycans, i.e., reduced terminal N-glycan processing, promoted cell invasiveness [10] and EGF-stimulated proliferation [8]. Although prior investigations have merited the role of N-glycosylation in cancer, additional studies are needed to further expose the influence of specific N-glycans in cancer progression.

Past studies from our lab using a rat *Mgat3* knockdown NB cell line, referred to as *Mgat3*-/-, with lessened bisected N-glycans yielded altered cell cycle progression, increased proliferation, and increased cell-cell adhesion relative to the parental NB_1 cells [23]. Here, using the BE(2)-C cell line, we further our prior studies to include a human mutant cell line with a CRISPR/Cas9 knockout of the *MGAT3* gene, resulting in functional loss of GnT-III. This newly generated cell line, (*MGAT3-/-*), had lowered bisected N-glycans relative to the parental cell line, as indicated via lectin blotting. Lectin blotting also suggested that oligomannose and β1,6-GlcNAc branched N-glycans were increased. Decreased levels of bisected N-glycans augmented non-adhered and adhered NB cell growth which coincided with alterations in the cell morphology and differentiation of *MGAT3-/-* cells. Likewise*, MGAT3-/-* cells displayed increased migration and invasion compared to BE(2)-C. Overexpression of GnT-III in both, human BE(2)-C and rat NB_1 cell lines supports that the higher levels of bisected N-glycans suppresses NB progression. The current study establishes that diminution of bisected N-glycans promotes NB development and progression.

## Materials and methods

### Cell lines and cell culture

Human BE(2)-C and rat B35 cell lines were acquired from American Type Culture Collection (Manassas, VA, USA). Clones were picked from the B35 cells to establish the NB_1 cell line and CRISPR/Cas9 technology was used to make the NB_1(*Mgat3*-/-) [10]. CRISPR/Cas9 technology was used to edit the gene Beta-1,4-Mannosyl-Glycoprotein 4-Beta-N-Acetylglucosaminyltransferase (*MGAT3*) (accession: CR45681) in BE(2)-C cells as previously described [10]. The CHOPCHOP web toolbox was used to design the sgRNAs [25–27]. The sgRNA oligonucleotide used to target *MGAT3* was CGGGACCTGAACTACATCCG (nucleotides 1,348−1,367). In brief, the pSpCas9(BB)-2A-Puro vector (Addgene plasmid ID: 48139) encoding *MGAT3* was used to create the GnT-III knockout in the BE(2)-C cell line, referred to as *MGAT3 -/-,* as previously described for other mutants [10]. Genomic fragment sequencing from an expanded NB cell clone revealed a 20-nucleotide deletion of the *MGAT3*, resulting in a frameshift mutation in the C-terminus region. The 20-nucleotide deletion was confirmed in nine separate bacterial cell clones. In short, bacterial cells were transformed with the amplified DNA fragment expressed in a TA cloning vector. In total, nine white bacterial colonies were sequenced to confirm the 20-nucleotide deletion. Cells were maintained at 5% CO_2_ at 37 °C in complete DMEM (10% FBS, 50 U/mL penicillin, 50 μg/mL streptomycin). Mouse Mgat3-pCDNA3.1-Hygromycin vector kindly provided by Dr. Pamela Stanley, College of Albert Einstein, was used for overexpression and rescue in cell lines as specified via Lipofectamine^®^ 3000 (Thermo Fisher Scientific, Rochester, MA, USA) protocol [10].

### Cell morphology

Cells were seeded at low density on CellBind Culture dishes (Corning, NY, USA) and incubated for 20 h followed by image acquisition using an Olympus CKX41 microscope (Olympus, Tokyo, Japan) equipped with a 40 × objective. Cells were categorized as I-type, S-type or N-type as described [28,29], and then the percentage of each cell type was recorded.

### Cell migration of dispersed cells

Cell migration was investigated using BD Falcon cell chambers (BD biosciences, CA, USA) as previously noted [10]. In brief, complete DMEM was placed into the wells of a 24 well dish, followed by placement of a trans-well insert in each well. Subsequently, dispersed cells (2.5 × 104) were placed into the trans-well insert and allowed to migrate for (19 hrs) at 37 °C. Migrated cells (those on lower portion of insert) were fixed with 100% methanol and stained with 1% Toluidine blue. Subsequently, the membrane was cut from the insert and affixed to a microscope slide for analysis. The number of migrated cells were counted using a Nikon TMS microscope. Images were obtained using an Olympus CKX41 microscope (Olympus, Tokyo, Japan) (20 × objective).

### 3D cell spheroid formation

3D cell spheroids were generated using SPHERICALPLATE 5D® (Kugelmeiers, Erlenbach, Sweden) as previously described [10]. In brief, 0.5 mL of DMEM media was added to wells of the plate and centrifuged (3 min/500 × *g*) to eliminate air bubbles. Cells were added (7.5 × 10^4^ per well) and spheroids allowed to form overnight (37 °C, 5% CO_2_) in complete DMEM (10% FBS, 50 U/mL penicillin, 50 μg/mL streptomycin).

### Whole cell lysates

Whole cell lysates were collected in RIPA buffer (PBS, 1% Triton X-100, 0.5% sodium deoxycholate, 0.1% SDS, protease inhibitor cocktail set III (EMD Biosciences, San Diego, CA, USA) as previously described [10]. In summary, cells were grown to confluency on 35 mm dishes and subsequently scraped off the dish and resuspended in RIPA buffer. Cells were sheared via a 20 G needle and then placed on ice for 30 min, followed by centrifugation (15,000 × *g* / 20 min / 4 °C; Eppendorf F-45–30−11 rotor (Eppendorf, Westbury, NY, USA)) and supernatant retrieved. Samples were stored at −80 °C until ready for use.

### Lectin blotting and coomassie-stained gels

Proteins were separated on 10% SDS-PAGE (Bio-Rad, Hercules, CA, USA) and transferred to a PVDF membrane (Millipore, Billercia MA, USA), as previously reported [10]. Membranes were blocked with 5% dry milk and the probed with biotin-conjugated lectins: *Phaseolus vulgaris* Erythroagglutinin (E-PHA), Galanthus Nivalis Lectin (GNL), or Phaseolus Vulgaris Leucoagglutinin (PHA-L) (Vector Laboratories, Burlingame, CA, USA). Membranes were washed three times (1X PBS + 1% Tween20), and then incubated with streptavidin (Vector Laboratories, Burlingame, CA, USA). Blots were developed with 1-Step™ NBT/BCIP (Thermo Scientific, Rockford, IL, USA). Lectin band intensities were measured using Image J software, version 1.54d.

### Cell dissociation

Cells were dissociated as previously described [10]. Briefly, cells were grown to confluency on CellBind Culture dishes (Corning, NY, USA), washed twice with DMEM, and then, using a cell scraper, detached from the dish. Subsequently, cells were dissociated by pipetting up and down using a 1 mL pipette tip. Microscopy images of cell clusters were obtained using an A IX71 Olympus microscope (Olympus, Tokyo, Japan) equipped with a 20 × objective. Area of cell clusters (<10 cells) was measured using Image J software, version 1.51.

### BrdU proliferation

Cell proliferation was measured by examination of 5-bromo-2-deoxyuridine (BrdU) internalization using the BrdU cell proliferation assay kit (Millipore, Billerica, MA) following the manufacturer’s protocol [10]. In brief, 2 × 10^4^ dispersed cells were pipetted into individual wells of a 96-well dish and incubated with BrdU reagent for 22 hrs under standard culturing conditions. Following incubation, cells were fixed for 30 min at room temperature, anti-BrdU monoclonal antibody added followed by secondary goat-α-mouse IgG peroxidase conjugate. Absorbance was measured at 450 nm using a Multiskan FC plate reader (Fisher Scientific, Atlanta, GA, USA), and calculated:


$$\begin{array}{c} {average\;ABS}_{+BrdU}-{average\;ABS}_{-BrdU}=Relative\;Rate\;of\;Proliferation. \end{array}$$

### Anchorage independent growth

Anchorage-independent growth was assessed using the soft agar assay, as indicated previously [10]. In summary, a mixture (1:1) of 1% noble agar and 2X DMEM was pipetted into a 35 mm dish and allowed to solidify. Next, a cell suspension (8.5 × 10^3^ cell/mL in DMEM) was mixed (1:1) with melted, cooled 0.6% noble agar, gently mixed, and pipetted into wells with previously solidified agar layer. Upon solidification of upper layer, 0.5 mL of DMEM was added on top of agar layers to avoid drying of agar layers. Plates were incubated and cell colonies allowed to form for 13 days. Post 13-day incubation, images were acquired using a IX71 Olympus microscope, and the area of the cell growth was measured using Image J software, version.

### 3D spheroid invasion

Cell spheroids (1 day formed) were collected from SPHERICALPLATE 5D® (Kugelmeiers, Erlenbach, Sweden) and placed in a 15 mL conical tube. Spheroids were allowed to settle, harvested and then pipetted into pre-cooled Matrigel (Corning, Corning, NY, USA), and gently mixed as previously noted [10]. The spheroid–matrigel mix (40 μL) was pipetted into a 24-well dish and incubated (37 °C) for 30 minutes to allow the Matrigel to solidify. Next, complete DMEM (1 mL) was added, and spheroids were allowed to invade for 20 hrs, 24 hrs, 91 hrs, or 120 hrs, as indicated. Images of spheroid invasion were acquired using an IX71 Olympus microscope (10 × objective). The invasion area and cell spheroid area were measured with Image J software, version 1.54d, and calculated:


$$\begin{array}{c} Invasion\;Area=Invasive\;Area-Spheroid\;Area \end{array}$$

### Data analysis

Adobe Photoshop was used to prepare images for Figs. Origin 9.55 was utilized for graphics and statistics. Data are reported as the mean ± standard error of the mean (SEM). Where shown, “*n*” denotes the number of observations. Unpaired Student’s *t*-test was used to compare two groups, while one-way ANOVAs with the post hoc Holm–Bonferroni means comparison test was used to compare more than two groups.

## Results

### Generation and characterization of the MGAT3-/- NB cell line

**Fig 1:**
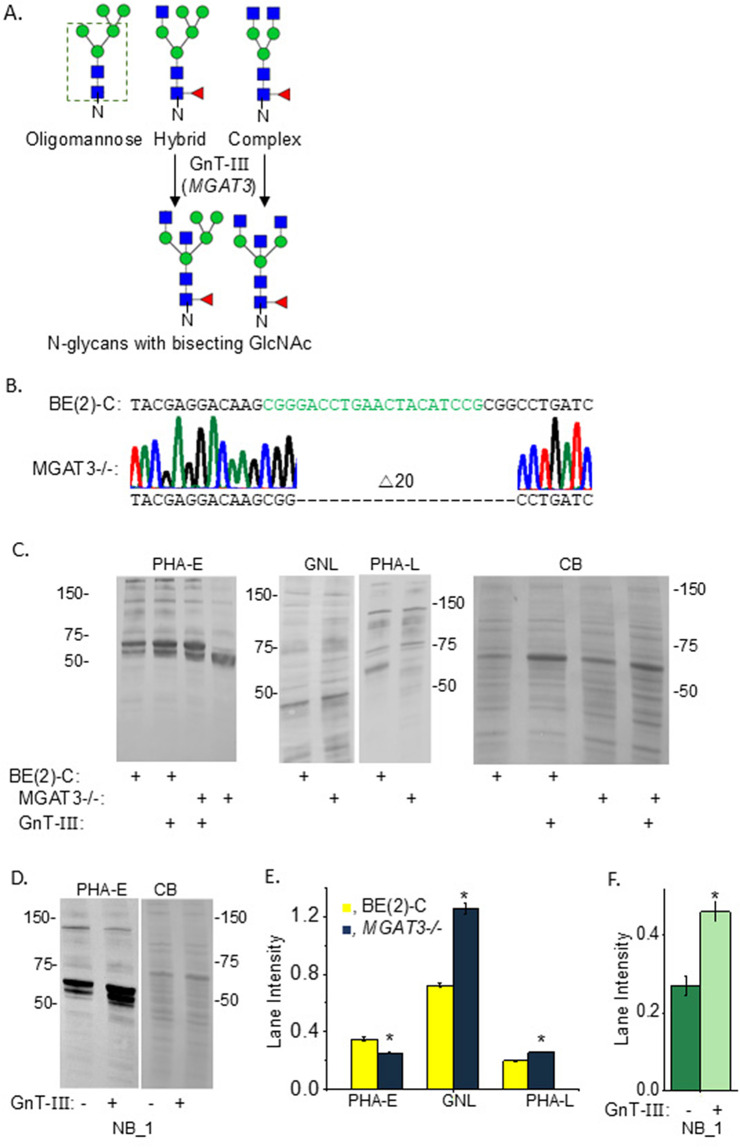
Engineering and characterization of the newly engineered *MGAT3-/-* NB cell line. The three general types of N-glycans and the addition of GlcNAc to the core Man of hybrid and complex N-glycans by GnT-III (*MGAT3*) **(A)**. GnT-III does not use oligomannosylated proteins as substrate. N denotes Asn residue of an N-glycosylated protein. The dashed blue box encircles the common pentasaccharide core. Blue and green symbols denote GlcNAc, and Man residues, respectively. Be(2)-C DNA sequence of a fragment of *MGAT3* containing the gRNA target sequence (green font), along with a DNA chromatogram of the same amplified fragment from *MGAT3-/-* cells with a deletion of twenty nucleotides (ρ20) **(B)**. Lectin blots and Commassie blue stained gel of proteins from whole cell lysates of human Be(2)-C cell lines **(C)** and the rat NB (NB_1) cell line **(D)**. Separated proteins were probed with Phaseolus vulgaris erythroagglutinin (PHA-E), Galanthus Nivalis Lectin (GNL), or Phaseolus Vulgaris Leucoagglutinin (PHA-L). Coomassie blue (CB) stained protein gels demonstrate equal protein loads among the compared samples. Numbers adjacent to the blots signify protein markers (in kDa) as indicated. Bar graphs denote total lane band intensities of lectin blots Be(2)-C and *MGAT3*-/- NB cell lines, **(E)** and the rat NB cell line (NB_1) transiently expressing GNT-III **(F)**. Student t-test was used for comparison of two samples at p < 0.01 (*).

There are three general types of N-glycans: oligomannose, hybrid, and complex (Fig 1A). GnT-III, encoded by *MGAT3*, can catalyze the addition of GlcNAC to the central mannose (Man) residue of the conserved pentasaccharide core of hybrid and complex types of N-glycans [13]. The addition of bisecting GlcNAc residues terminate the processing of N-glycans [18]. To investigate the role of this modification, CRISPR/Cas9 technology was employed to introduce a frameshift mutation in *MGAT3* of the human NB cell line, Be(2)-C. An amplified genomic DNA fragment of *MGAT3* from genomic DNA of an expanded cell colony revealed a 20-nucleotide deletion (Fig 1B). This same deletion nucleotide sequence was identified in eight additional bacterial colonies. The human parental and mutant cell lines are referred to as BE(2)-C and *MGAT3*-/-, respectively, throughout the text.

To validate functional loss of GnT-III, we applied lectin binding studies of whole cell lysates from BE(2)-C and *MGAT3*-/- cell lines. Separated proteins from whole cell lysates of BE(2)-C and *MGAT3-/-* cells were probed with PHA-E, a lectin which binds with high affinity to bisecting GlcNAc N-glycans of complex type [30] (Fig 1C). PHA-E binding was greatly diminished in *MGAT3-/-* relative to BE(2)-C. Further when both BE(2)-C and MGAT3-/- cell lines were transiently transfected with *Mgat3*, levels of bisecting GlcNAc N-glycans were significantly increased in the cell lines. Lectin blotting was also performed with GNL and PHA-L which binds tightly to oligomannose and β1,6-GlcNAc branched N-glycans, respectively [30]. In both cases, lane intensities were higher in samples of the *MGAT3*-/- cell line than in BE(2)-C. Previously, we showed that levels of bisecting GlcNAc N-glycans were quite low in a rat NB cell line, NB_1, relative to the human BE(2)-C cell line [23]. When GnT-III was overexpressed in the NB_1 cell line there was a considerable increase in the level of bisecting GlcNAc N-glycans (Fig 1D). In all cases, the Coomassie blue-stained gel (CB) showed that samples had similar levels of protein per lane. To support the representative lectin blots, total lane intensities of several lectin blots were normalized to total lane intensities of coomassie blue-stained gels for comparison of BE(2)-C to *MGAT3*-/- (Fig 1E), and NB_1 with or without overexpression of *Mgat3* (Fig 1F). Thus, the identified frameshift mutation in *MGAT3*-/- causes a reduction in bisecting GlcNAc N-glycans, and furthermore, the *MGAT3-/-* cell line was rescued by overexpression of GnT-III. These results indicate that GnT-III activity was significantly lowered in the *MGAT3-/-* cell line. Moreover, the results supported that oligomannose and β1,6-GclNAc branched N-glycans were higher in the *MGAT3*-/- cell lines relative to the BE(2)-C cell line.

### Decreased levels of bisected N-glycans enhanced non-adhered and adhered NB cell growth

**Fig 2:**
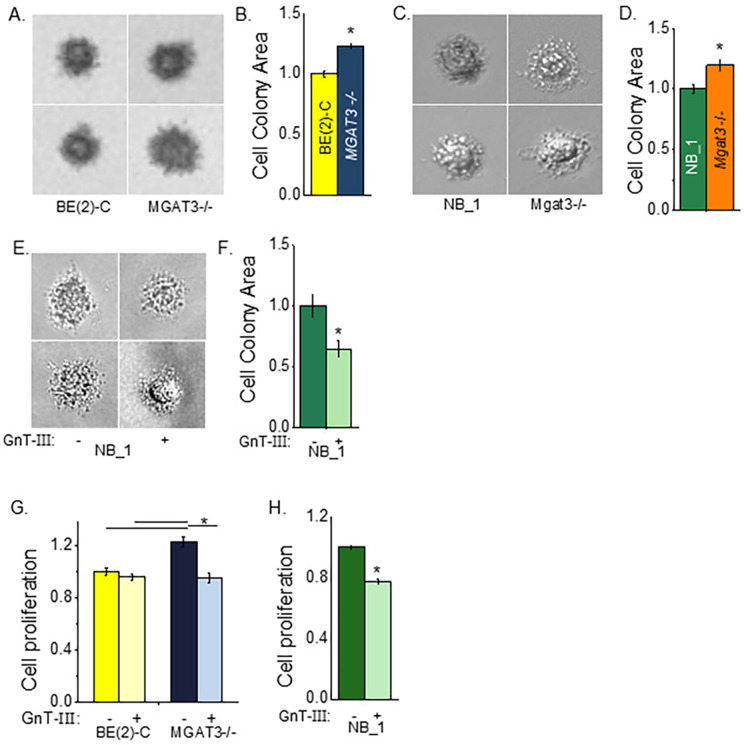
Adhered and non-adhered cell growth of the parental and N-glycosylation mutant cell lines. Representative micrographs of cell colonies **(A)** and the average area of cell colonies of BE(2)-C (*n* = 464) and *MGAT3*-/- (*n* = 465) cell lines at 13 days of growth **(B)**. Micrographs **(C)** and cell colony size **(D)** of rat NB_1 (*n* = 378) and *Mgat3*-/- (*n* = 387) cells. Images **(E)** and colony size **(F)** of rat NB_1 cells without (-; *n* = 59) and with (+; *n* = 46) GnT-III. Images were acquired using a IX71 Olympus microscope at 4× (panel **A)**, 10× (panel **C)**, and 20× (panel **E)**. *n* denotes the number cell clusters. Cell proliferation of BE(2)-C *(n =* 9) and *MGAT3-/- (n =* 5) without and with GnT-III **(G)** and rat NB_1 cells (*n* = 10) without and with GnT-III **(H)**. *n* is the number of wells. In all cases, data was normalized to the parental cell line and shown as mean ± SEM. Student t-test for comparison of two samples and one way ANOVA with Holm-Bonferroni mean comparison of three or more samples at p < 0.01 (*).

Anchorage-independent cell growth was used to ascertain the aggressiveness of cells to grow in a non-adhered manner. After 13-days of cell growth, images were obtained and analyzed, and the colony size of the *MGAT3*-/- cell line was substantially increased relative to the BE(2)-C cell line (Fig 2A and 2B). An increase in non-adhered cell growth for 12-days was also observed for the rat *Mgat3-/-* cell line compared to the rat parental cell line NB_1 (Figs 2C and 2D). To demonstrate that higher levels of bisected N-glycans suppress non-adhered cell growth in NB cells, images were acquired of the rat NB_1 cell line without and with overexpression of GnT-III after 13-days of non-adhered growth (Fig 2E). Quantification of the area colonies (Fig 2F) showed that increased levels of bisected N-glycans significantly reduced anchorage-independent cell growth. The level of BrdU incorporated into the DNA during its replication process on cell culture plates at about 75% confluency was employed to evaluate cell-attached proliferation. Cell proliferation occurred at a faster rate in the *MGAT3*-/- cell line than the BE(2)-C cell line (Fig 2G), and furthermore, overexpression of *Mgat3* decreased cell proliferation in the *MGAT3-/-* cell line. Additionally, when *Mgat3* expression was increased in the rat NB_1 cell line, cell proliferation declined (Fig 2H). Thus, both non-adhered and adhered cell growth in distinct NB cell lines occurred more rapidly when bisected N-glycans levels were lowered and occurred more slowly when bisected N-glycans were raised. The results also supported that the faster proliferation rate in the *MGAT3*-/- cell line was due to lowered GnT-III activity.

### Decreased levels of bisected N-glycans modified NB cell morphology

**Fig 3:**
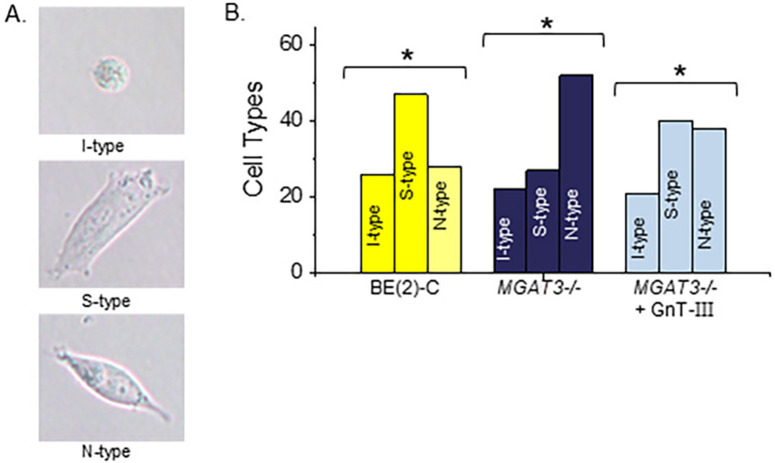
Cell morphological differences between *MGAT3*-/- and BE(2)-C. Representative images **(A)** of the various cell types as indicated, along with bar graph **(B)**, denoting the percentage of cell types observed in BE(2)-C (*n* = 74), *MGAT3*-/- (*n* = 74) and *MGAT3*-/- + GnT-III (*n* = 74), where *n* is the number of fields. Chi square test reported an association of cell lines with cell types at p = 0.01 (*).

Three different cell types were observed in the BE(2)-C, *MGAT3*-/-, and overexpression of GnT-III in MGAT3-/- cells, including I-type, S-type, and N-type (Fig 3A). The *MGAT3-/-* cell line, which had the lowest levels of bisected glycans, showed significant differences in cell types relative to the BE(2)-C cell line (Fig 3B). BE(2)-C had the highest proportion of S-type cells while *MGAT3-/-* had the highest proportion of N-type cells. When *MGAT3-/-* cells were transiently transfected with *Mgat3* cDNA, the proportion of S-type cells was increased while the proportion of N-type cells was decreased, indicating that the difference in cell morphology was due to the level of bisected N-glycans. Hence, these studies reveal that modifying levels of bisected N-glycans modify the cell morphology and differentiation of NB cells.

### Strengthened cell-cell adhesion by decreased levels of bisected N-glycans

**Fig 4:**
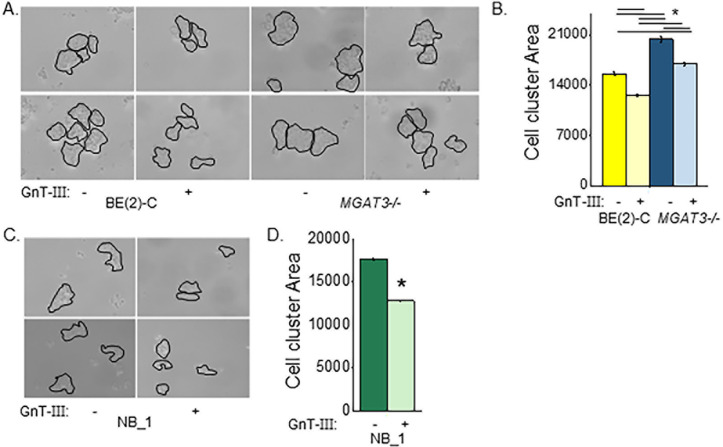
Varied GnT-III activity impacts cell-cell adhesion. Typical DIC images of intact cell clusters **(A)** and average area **(B)** of cell clusters from BE(2)-C (*n* = 200), BE(2)-C + GnT-III (*n* = 340), *MGAT3-/-* (*n* = 221), and *MGAT3-/-* + GnT-III (*n* = 258) following mechanical dissociation via pipetting of cell monolayers. Data are shown as mean ± SEM. One way ANOVA followed by Holm-Bonferroni mean comparison of three or more samples at p < 0.01 (*). Micrographs **(C)** and corresponding bar graph **(D)** showing cell dissociation of rat NB_1 cells without (*n* = 258) and with GnT-III (*n* = 444). Samples were compared via student t-test at p < 0.0001 (*). In all cases, the *n* represents the number of cell clusters.

The dissociation of cell monolayers into cell clusters was employed to measure the adhesiveness of cells to each other. Representative micrographs of cell clusters in BE(2)-C and *MGAT3-/-* cell lines, along with those transiently transfected with *Mgat3* cDNA (Fig 4A). The cluster area significantly increased by about 1.3-fold when GnT-III activity was considerably reduced relative to the BE(2)-C cell line (Fig 4B). In both BE(2)-C and *MGAT3-/-* cell lines overexpressing GnT-III there were significant reductions in the cell cluster area. The rat NB_1 cell line overexpressing GnT-III also showed a marked decrease in the cell cluster area (Fig 4C and 4D). Thus, changes in the levels of bisected N-glycans modify the strength of cell-cell adhesion in NB cells.

#### NB cell migratory rates are increased by reduced levels of bisected N-glycans.

**Fig 5:**
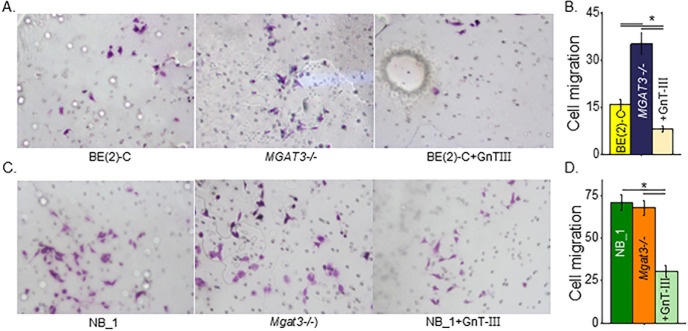
Lowered GnT-III activity increased cell migratory rates. Representative micrographs **(A)** and the number of migratory cells per field **(B)** from BE(2)-C, *MGAT3*-/-, and *MGAT3*-/- + GnT-III cell lines. Typical images **(C)** of rat NB_1, NB_1(*Mgat3*-/-) and NB_1 transfected with *Mgat3*, along with a bar graph of the number of migratory cells per field **(D)**. In all cases, data are shown as mean ± SEM, *n* = 16, where *n* denotes the number of fields. One-way ANOVA with Holm-Bonferroni mean comparison was used to compare samples at *p < 0.05.

Dispersed cells on membrane inserts were allowed to migrate from upper to lower chambers for 19h. The number of migratory cells were scored on four fields per slide. Representative micrographs of a field for BE(2)-C, *MGAT3-/-*, and BE(2)-C overexpressing GnT-III (Fig 5A) and quantification of migratory cells for each line (Fig 5B). NB cells with reduced GnT-III activity had markedly increased migratory rates relative to BE(2)-C, while overexpression of GnT-III significantly slowed cell migration. To show a general trend in NB cells when MGAT3 expression is altered, a similar migratory assay was performed with the NB_1 and *Mgat3*-/- cell lines. Quantification of obtained images (Fig 5C) showed that increased expression of GnT-III in the rat NB_1 cells greatly reduced cell migratory rates while lowered GnT-III was not significantly different (Fig 5D). These results indicate that increased activity of GnT-III decreases migratory rates in NB cells while lowered activity of GnT-III has an opposing effect on migratory rates.

### Lowered levels of bisected N-glycans promote NB cell invasiveness

**Fig 6:**
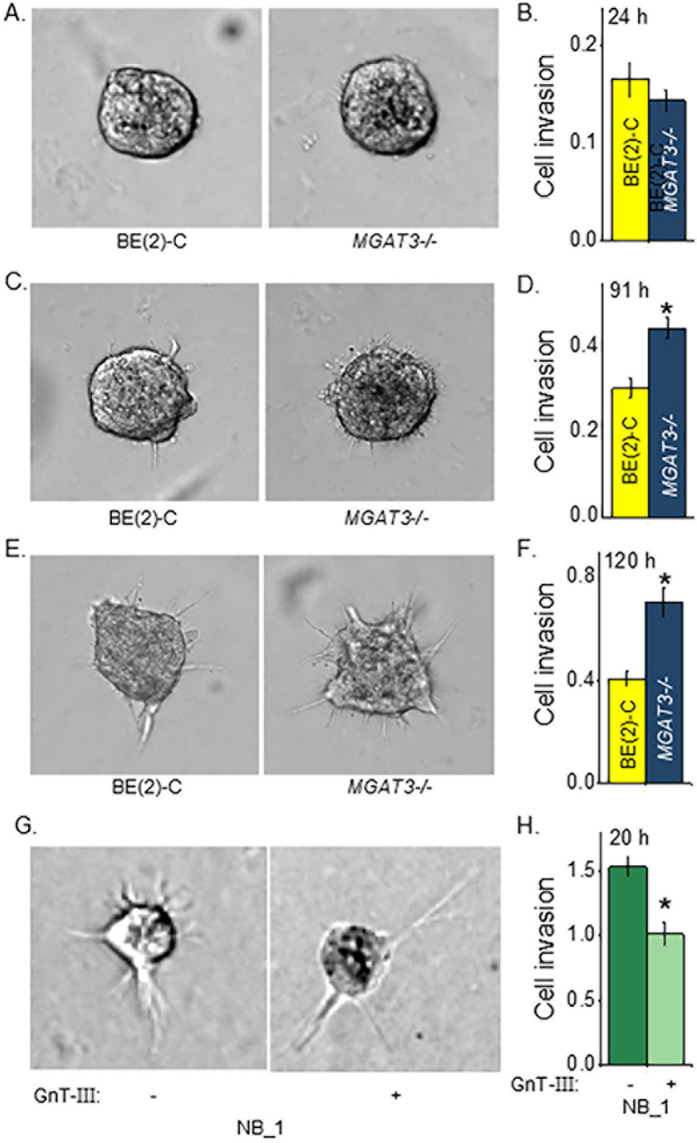
Lowered GnT-III activity enhanced cell invasion rates. Images and the quantification of the area of invasion from BE(2)-C and *MGAT3*-/- cell lines at 24 h (*n* = 30 and 36) **(A, B)**, 91 h (*n* = 42 and 42) **(C, D)**, and 120 h (*n* = 49 and 50) **(E, F)**. Micrographs **(G)** of rat NB_1 and NB_1 transfected with *Mgat3*, along with a bar graph denoting area of invasion after 20 h (*n* = 21 and 21) **(H)**. Data are shown as mean ± SEM, where *n* denotes the number of invasive spheroids. *p < 0.0001.

The ability of cell spheroids to invade and migrate through an extracellular matrix was measured. Micrographs and quantification of BE(2)-C and *MGAT3-/-* cell spheroids invading for 24 h (Fig 6A and 6B), 91 h (Fig 6C and 6D), and 120h (Fig 6E and 6F) showed that deceased *MGAT3* expression promoted cell invasiveness at the latter two time points, and a general trend that cell invasiveness increased with increased invasion time. To illustrate how overexpression of GnT-III decreases cell invasiveness, the invasion of rat NB_1 without and with overexpression of GnT-III were analyzed (Fig 6G and 6H). Rat NB_1 cells with GnT-III overexpression were significantly less invasive following 20 h. Thus, reduced GnT-III activity increases 3D invasiveness and increased GnT-III activity suppresses 3D invasiveness in NB cell spheroids.

## Discussion

**Fig 7:**
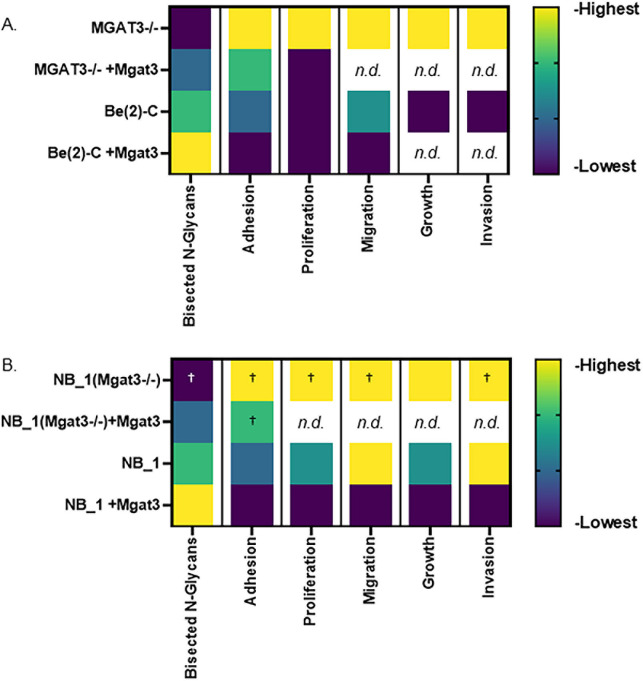
Similar phenotypic trends in human and rat NB cells due to varied GnT-III (*MGAT3*) activities. Heatmap ranking the relative bisecting N-glycan expression and cellular phenotypes, including adhesion, proliferation, migration, growth, and invasion, in human BE(2)C and *MGAT3-/-* NB cell lines, along with those overexpressing GnT-III (*MGAT3-/-* + *Mgat3* and BE(2)C +*Mgat3*) **(A)**, and in rat NB_1 and NB_1(*Mgat3-*/-), plus those overexpressing GnT-III (NB_1(*Mgat3-/-)* +*Mgat3* and NB_1 + *Mgat3*) **(B)**. Where *†* represents data from Hall MK *et al*., 2023 and *n.d.* denotes not determined. Color scale represents relative ranking from highest (yellow) to lowest (dark blue).

Our lab has performed extensive investigations on the role of N-glycosylation in the progression of NB [8,10,12,23]. In this study, we continue our work by further detailing the influential role of GnT-III on NB growth, cell-cell adhesion, invasion, and migration. By first creating a CRISPR/Cas9 knockout of *MGAT3* in human BE(2)-C cells, we showed that functional loss of GnT-III causes a decline in the levels of bisected N-glycans. Moreover, lectin blotting suggested β1,6-GlcNAc branched N-glycans were raised as previously reported [18]; however, a surprise was the increase in oligomannose type N-glycans. Additionally, the functional loss of GnT-III was associated with increased adhered cell proliferation, non-adhered cell growth, cell invasion, cell-cell adhesion, and cell migration. Moreover, non-adhered cell growth was increased in a rat NB (NB_1) cell line with lowered GnT-III (*Mgat3-/-*), as was cell proliferation, and adhesion [23]. Upon rescue of GnT-III by transient expression of a mouse GnT-III cDNA, which increased levels of bisected N-glycans, we observed a phenotypic rescue, reducing the adhered growth, and adhesion of *MGAT3-/-* cells, and a similar trend in cell adhesion was reported in the rat NB cell line [23]. Further overexpression of GnT-III in BE(2)-C and NB_1 cells decreased cell adhesion and migration, along with cell growth, proliferation and invasion in the NB_1 cell line. Cell morphology analysis of *MGAT3-/-* cells revealed an increase in N-type cells and a decrease in S-type cells compared to BE(2)-C cells while the I-type cells were quite similar. Further, reintroduction of GnT-III increased the level of S-type cells. We suspect that S-type cells account for the slower proliferation rates in BE(2)-C and *MGAT3-/-* overexpressing GnT-III since S-type cells have much slower proliferation rates than I- and N-types of cells [28,29]. These results, along with our previous study [23] provide a thorough understanding of how *MGAT3* expression impacts NB as highlighted by the directional changes in cellular phenotypes with respect to the levels of bisected N-glycans, as summarized in Fig 7. Thus, the consistent findings using two NB cell lines strongly support that reduced *MGAT3* expression is associated with aggressive phenotypes, while increasing *MGAT3* expression deters aggressive NB.

The role of bisected N-glycans in NB progression was compared to cellular phenotypes in rat NB_1 cells, which have very low levels of bisected N-glycans as shown by ESI-MS and MALDI TOF MS, along with lectin blotting [23,31]. This low level is supported by the lack of difference in cell invasiveness and migration between rat *Mgat3-/-* and parental NB_1 cell lines [23]. However, it should be noted that increased levels of bisected N-glycans by overexpression of GnT-III in NB_1 did slow cell migration and invasiveness, as shown here. Moreover, this increase in GnT-III activity hindered cell proliferation, non-adhered cell growth, and cell-cell adhesion. Based on lectin blots, it appears there are much higher levels of bisected N-glycans in the Be(2)-C cell line than the NB_1 cell line [23]. This higher level is supported by the findings that overexpression of GnT-III in Be(2)-C did not significantly decrease cell proliferation while lowered levels of bisected N-glycans increased cell growth. Taken together, these results clearly support that increased levels of bisected N-glycans are related to NB with lessened aggressive phenotypes, and furthermore, argue that increased levels of bisected N-glycans may result in NB regression.

GnT-V has often been associated with the malignant transformation of cells [4,32–34]. Because GnT-V and other glycosyltransferases prefer N-glycans without bisecting GlcNAc as substrate [18], GnT-III has often been considered a tumor suppressor [19,22]. However, as discussed by Taniguchi et al., 2021 [33] GnT-III activity has been found to be increased in splenic, hepatic and ovarian cancer. In neuroblastoma, overexpression of GnT-III stalled migration, while knockdown increased migration, likely stemming from direct modification of α3 integrin [19]. GnT- III activity is closely regulated with raised levels during cell growth, and reduction upon neuronal differentiation of GOTO NB cells [24]. Our current study showed that lowered GnT-III levels enhanced cell migration and decreased the number of highly proliferating cell types. A previous study also found that lowered levels of GnT-III, and thus bisected N-glycans, increased the percent of cells in the G1-S transition, and thus cell proliferation in rat NB_1 cells [23]. Furthermore, we have shown here that loss of GnT-III promotes NB invasiveness while increased expression of GnT-III represses NB invasiveness in 3D cell culture. While the role of GnT-III in cancer biology may be conflicting, our findings strongly support that raised expression of bisected N-glycans represses NB progression.

A striking finding was that oligomannose type N-glycans were increased in the *MGAT3*-/- cell line. Previously, we showed that the cell invasiveness was greatly increased when GnT-I activity was lowered and oligomannose N-glycans were increased in rat NB_1 cells, a clone of rat B35 NB cells [10], as well as human Be(2)-C cells [8]. Further the rat NB_1 cell line with lowered *Mgat2* expression had lower levels of oligomannose than the parental cell line as determined by both MALDI and ESI MS techniques [23,31], and was observed to be significantly less invasive [10]. Here we demonstrated that the *MGAT3*-/- cell line has more oligomannose N-glycans and is much more invasive than the BE(2)-C cell line. These biology-driven results support our hypothesis that oligomannose type N-glycans are directly linked to cell invasiveness of NB. It is also a likely occurrence in other cancers as several studies have reported oligomannose N-glycans in several late-stage/advanced cancer types [35–40].

Numerous studies have linked higher levels of β1,6-branched N-glycans to aggressive and metastatic cancers [4,32–34]. In NB, higher levels of several β1,6-branched N-glycan structures were detected in cells derived from high-risk NB (NLF) relative to cells from low-risk NB (SY5Y) [41]. Further NLF cells are known to grow at a much faster rate than SY5Y cells [42,43]. Here the *MGAT3*-/- cells grew at a faster rate in adhered and non-adhered cell cultures compared to BE(2)-C cells; furthermore, the mutant cell line had higher amounts of β1,6-branched N-glycans. Together, these data support a vital role of β1,6-branched N-glycans in promotion of cell growth in NB. We conclude, it is vital to examine the levels of oligomannose and β1,6-GlcNAc branched N-glycans as the activity of GnT-III is changed in NB, as well as other cancers.

Our previous study of BE(2)C (*-MGAT1*) cells showed that oligomannosylated EGFR exhibited higher levels of autophosphorylation and ligand independent phosphorylation than BE(2)-C cells [8]. Various cell lines with altered expression of GnT-III impacted EGFR signaling; however, it was unclear how these changes in GnT-III expression altered N-glycans of EGFR. It was shown knockdown of GnT-III greatly enhanced EGFR phosphorylation, while overexpression of GnT-III decreased EGF affinity for EGFR in rat PC12 pheochromocytoma cells [44]. Furthermore, overexpression of GnT-III in MDA-MB-231 breast cancer cells led to decreased EGFR autophosphorylation and ERK signaling [45]. EGFR enriched with oligomannose N-glycans and EGFR with more terminal N-glycan processing are both more readily activated [8,44,45]. A future interest would be to identify the N-glycan structures attached to EGFR in *MGAT3*-/- NB cells and explore the impact of EGF-stimulated proliferation.

Here the results, along with the cited literature support that altering GnT-III alters NB growth and invasiveness. However, the limitations of this study are that a single *MGAT3-/-* cell clone was used. As such, testing for clonal variability was lacking but the results using rat NB_1 cell line revealed similar trends in cellular properties, see Fig 7. Secondly, lectin studies did support increased oligomannose N-glycans and lowered bisecting complex N-glycans in the *MGAT3*-/- cell line relative to the BE(2)-C cell line. However, detailed glycomics analysis would directly identify the N-glycan structures and the percents of oligomannose and bisected complex N-glycans and thereby strengthen these results.

## Conclusion

Herein we advance our prior investigations on the influence of disruptions in N-glycosylation on NB aggression by demonstrating how loss of GnT-III, and thus lessened bisected N-glycans, promotes NB growth, cell-cell adhesion, invasion, and migration. Moreover, we suggest that the diminution of bisected N-glycans coincides with an increase in both oligomannose and β1,6-branched N-glycans. Since oligomannose and β1,6-branched N-glycans have been implicated with enhanced cell invasiveness and growth, we emphasize the relevance of future studies to define roles of both N-glycans in response to modifying GnT-III activity and bisected N-glycans in NB progression.

## Supporting information

S1 File Gels and lectin blots. (PDF)

S2 File Data groups. (XLSX)
