# Supplementary material for: Neuroblastoma cell growth and invasiveness is modulated by the activity of N-acetylglucosaminyltransferase-III
Source: PLoS One. 2026 Jun 8;21(6):e0350822. doi: 10.1371/journal.pone.0350822 (PMC13245742; doi:10.1371/journal.pone.0350822)
Supplement: S1 File — (PDF) [file pone.0350822.s001.pdf]

CB

CB

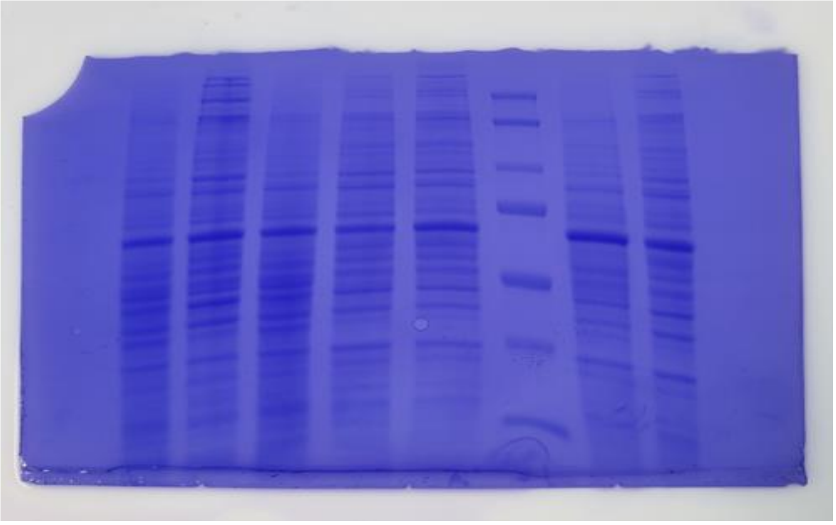

250801_RAS

NB_1
NB_1 + GnT-III
NB_1
BE(2)_C + GnT-III
BE(2)_C
Mgat3 -/-
(Mgat3 -/-) + GnT-III

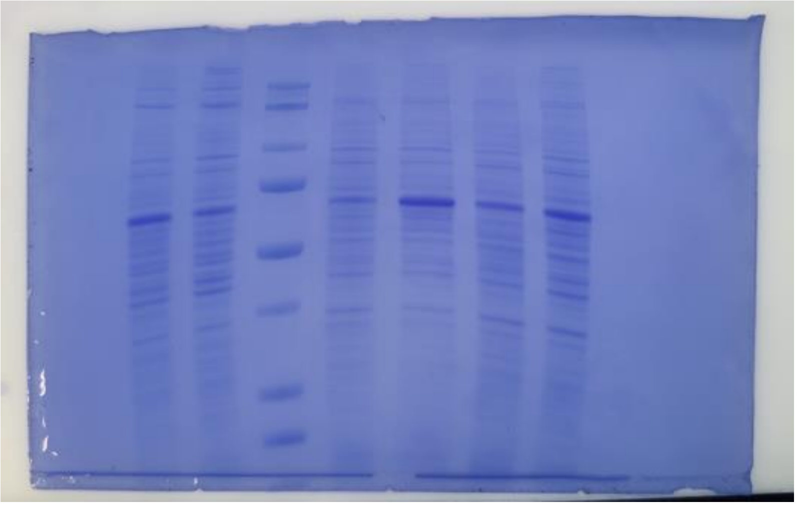

250825_mkh

NB_1
NB_1 + GnT-III
BE(2)_C + GnT-III
BE(2)_C
Mgat3 -/-
(Mgat3 -/-) + GnT-III

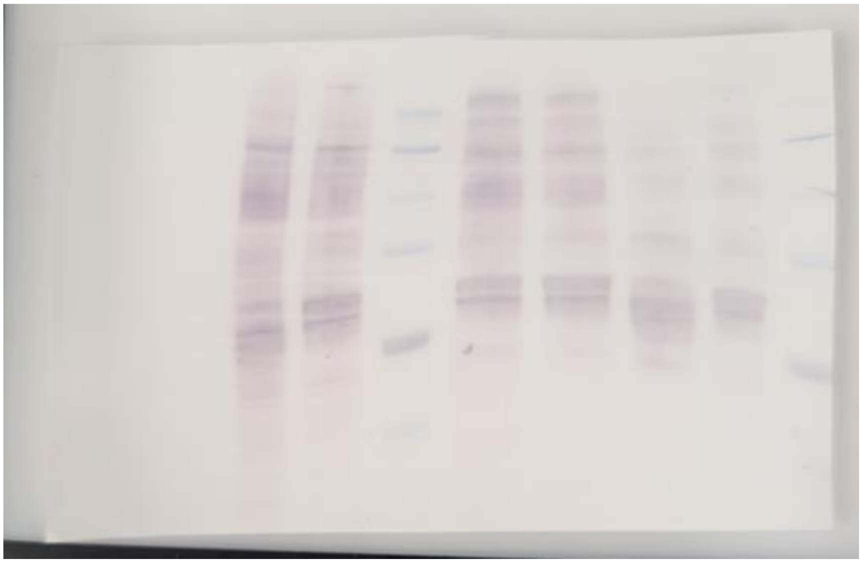

E-PHA

250804_MKH

NB_1
NB_1 + GnT-III
BE(2)_C + GnT-III
BE(2)_C
Mgat3 -/-
(Mgat3 -/-) + GnT-III

E-PHA

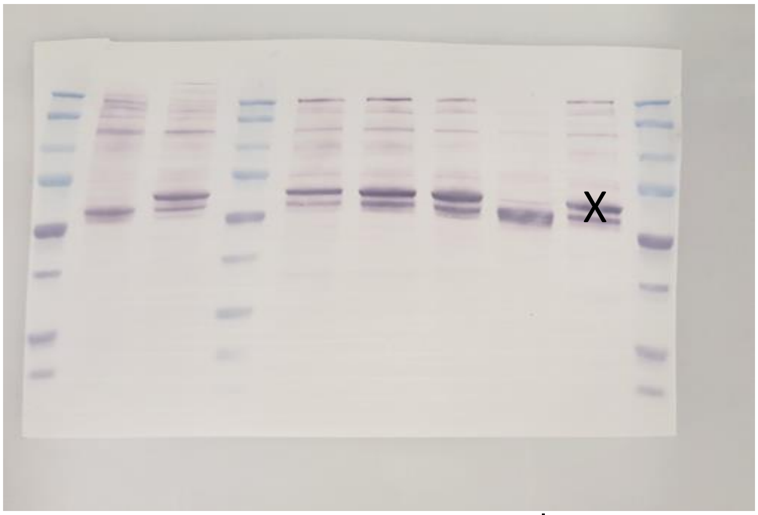

250812_mkh

NB_1 + GnT-III
NB_1
BE(2)_C
BE(2)_C + GnT-III
(Mgat3 -/-) + GnT-III
Mgat3 -/-

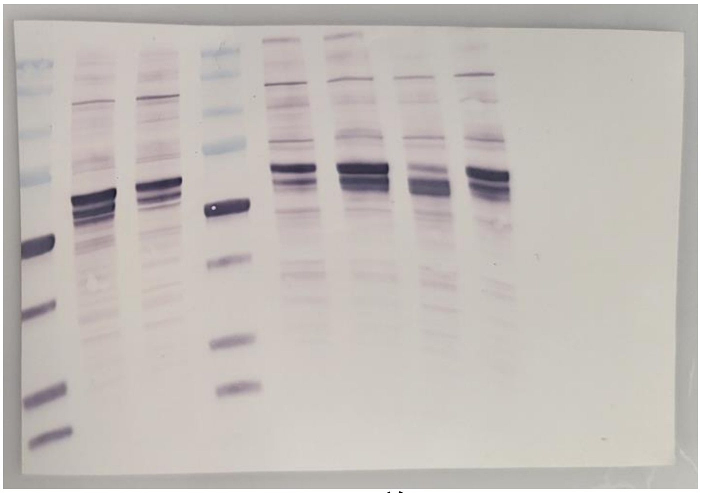

E-PHA

250827_MKH

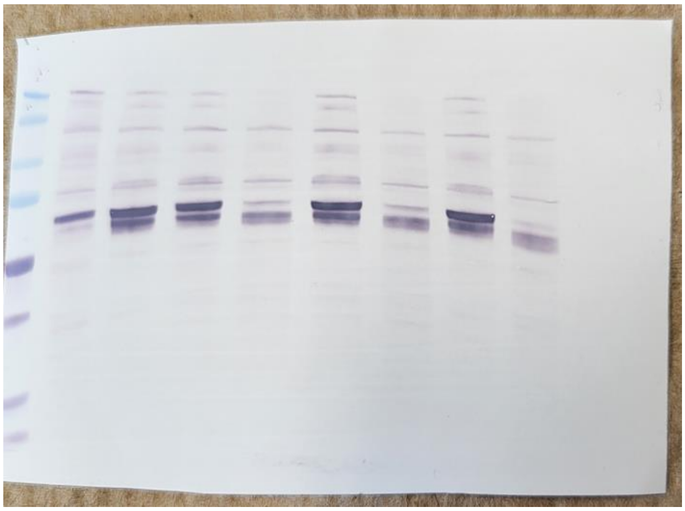

E-PHA

250829_MKH

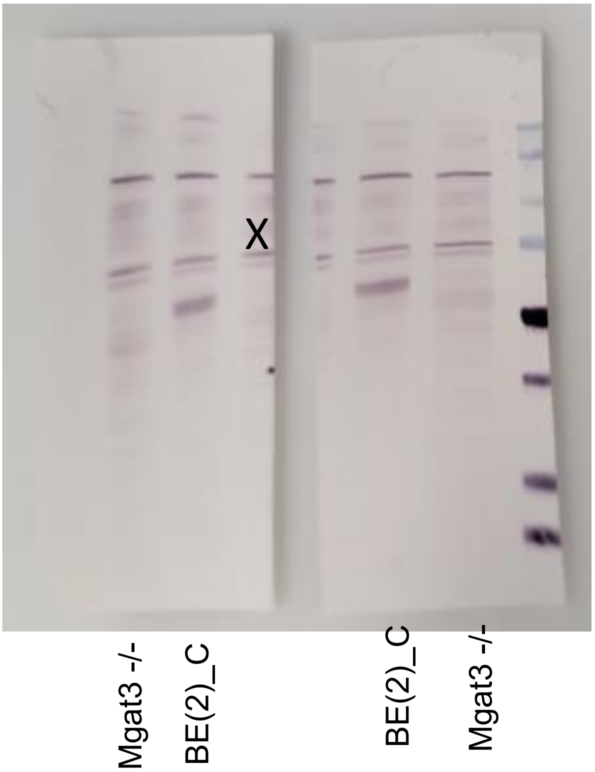

L-PHA

250925_mkh

Mgat3 -/- BE(2)_C BE(2)_C Mgat3 -/-

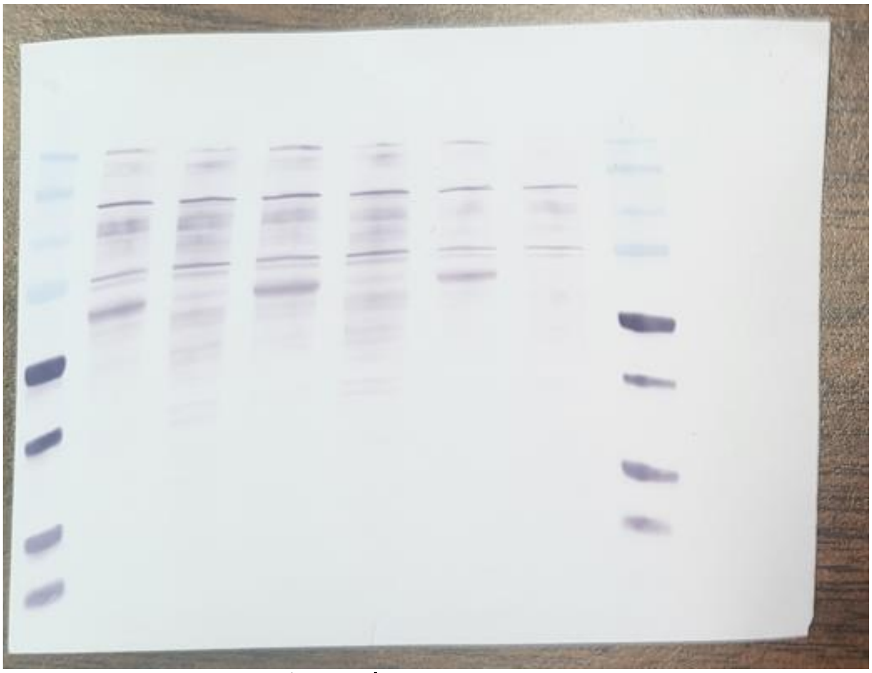

L-PHA

251002_mkh

BE(2)_C Mgat3 -/- BE(2)_C Mgat3 -/- BE(2)_C Mgat3 -/-

GNL

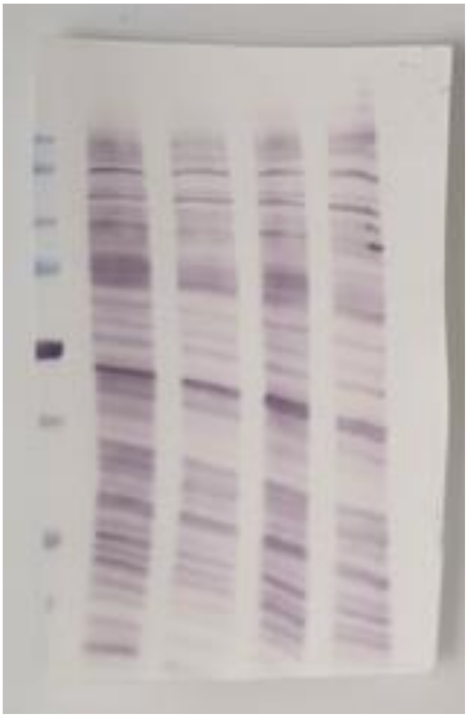

250925_mkh

Mgat3 -/- BE(2)_C Mgat3 -/- BE(2)_C

250930_mkh

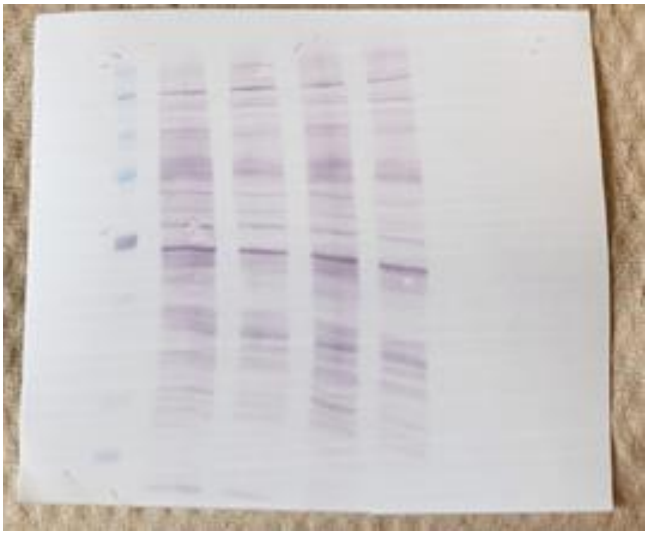

Mgat3 -/- BE(2)_C Mgat3 -/- BE(2)_C
