# Supplementary material for: Neuroblastoma cell growth and invasiveness is modulated by the activity of N-acetylglucosaminyltransferase-III
Source: PLoS One. 2026 Jun 8;21(6):e0350822. doi: 10.1371/journal.pone.0350822 (PMC13245742; doi:10.1371/journal.pone.0350822)
Supplement: Attachment — Submitted filename: Response to reviewers.pdf [file pone.0350822.s005.pdf]

POINT-BY-POINT RESPONSE TO REVIEWERS

Reviewer #1: After addressing the following two points, this manuscript could be accepted for publication in PLOS ONE.

Line 45: please revise the wording around “modifying N-glycan expression” for clarity.

Authors’ response: We revised this sentence to specify the relevant genes and clarify that changes in glycosyltransferase expression alter N-glycan branching (number and elongation of branches).

Please use consistent terminology when referring to the human BE(2)-C neuroblastoma (NB) and BE(2)-C cell lines throughout the manuscript.

Authors response: This has been addressed throughout the manuscript. We have used the BE(2)-C cell line.

Reviewer #2: Overall, the authors have addressed most of the concerns raised. However, the manuscript would be strengthened by a more explicit discussion of study limitations. In particular, please note (1) the lack of comprehensive glycomics analysis and (2) the potential impact of clonal variability, given that the knockout analysis is based on a single clone. Clearly stating these limitations will better contextualize the conclusions and strengthen the rigor of the study.

Authors response: This concern has been addressed, see last paragraph in the discussion section.
