# Supplementary material for: Neuroblastoma cell growth and invasiveness is modulated by the activity of N-acetylglucosaminyltransferase-III
Source: PLoS One. 2026 Jun 8;21(6):e0350822. doi: 10.1371/journal.pone.0350822 (PMC13245742; doi:10.1371/journal.pone.0350822)
Supplement: Attachment — Submitted filename: 260408_RESPONSE TO REVIEWERS.docx [file pone.0350822.s004.docx]

**POINT-BY-PONT RESPONSE TO REVIEWERS’**

**Reviewer #1:** In this manuscript, Burch et al. reports the study on the influential role of GnT-III on NB growth, cell-cell adhesion, invasion, and migration. GnT-III has long been considered as a tumor suppressor. This study created human MGAT3 knockout NB cells, and found the functional loss of GnT-III caused a decline of bisected N-glycans, and increase of β1,6-GlcNAc branched and oligomannose type N-glycans. The results suggest the importance of GnT-III activity in NB cell growth and invasiveness. The experimental details and characterization are well-documented, and the subject area and findings are of considerable importance. It is sound for publication in the PLOS One, however, a few points should be addressed before acceptance.

In text and figures, the refer of cell lines, including the wt and knockout cells are somehow not clear. It is suggested to clarify the description (BE2-C or NB-1, wt or knockout or GnT-III complementary).

**Authors:** We have changed Wt to Be(2)-C and checked the wording throughout to clarify names of cell lines. Since Be(2)-C is a human cell line the mutant cell line is noted as MGAT3-/- while the NB_1 cell is a rat NB cell lines the mutant is referred to as Mgat3-/-.

A shematic diagram of the correlation among bisected N-glycans, β1,6-GlcNAc branched and oligomannose type N-glycans could be provided to benefit the understanding of this work.

**Authors:** Since Glycomic analysis was not completed, we have concluded the reduced GnT-III activity (or lowered bisecting N-glycans) increased cell invasiveness, growth, migration, and cell-cell adhesion. The results from this study and our past study strongly support our conclusion that similar phenotypic trends in NB cells are observed as GnT-III (MGAT3) activities are varied, see our added figure, Fig 7. We have speculated the involvement of oligomannose and β1,6-GlcNAc branched N-glycans in altering these cellular phenotypes. See edits in abstract and throughout text of manuscript which can easily be viewed in marker version of the manuscript. Our future goal is to obtain funding to conduct Glycomic analysis.

**Reviewer #2:** This manuscript examines the effects of MGAT3/GnT-III deficiency on neuroblastoma cell behavior using a human BE(2)-C MGAT3 knockout model together with complementary experiments in a rat NB_1 system. The topic is interesting, and the phenotypic dataset is relatively broad, including proliferation, anchorage-independent growth, migration, spheroid invasion, cell-cell adhesion, and morphology. Overall, the study has merit and may become suitable for publication after major revision. However, in its current form, several mechanistic conclusions are stronger than the data support.

1. At present, the conclusions rely heavily on lectin blotting, which is useful but does not allow a sufficiently precise definition of the underlying structural changes. In particular, the current data do not clearly distinguish whether the observed phenotypes are attributable specifically to the loss of bisecting GlcNAc itself, to altered β1,6 branching, to changes in sialylation, or to broader remodeling of other glycan structures. More generally, it also remains unclear whether the phenotypes should be interpreted specifically in terms of N-glycan alterations alone. Because MGAT3 deficiency would be expected to affect glycan processing more broadly, the authors should avoid assigning the observed phenotypes too directly to a single glycan feature unless additional structural evidence is provided. Ideally, this point should be addressed by more detailed glycomics analyses. Alternatively, a broader multi-panel lectin blot analysis would at least help to define the glycan changes more comprehensively.

**Authors:** This study related the phenotypes to various types of glycans, not a single glycan structure. We do agree that glycomic analysis is needed to strongly support that oligomannose and β1,6-GlcNAc branched N-glycans are raised in the MGAT3-/- cell line and thus contributing to cell invasiveness and growth, respectively. We also wish to add the following for clarification purposes. Since the genetic modification in MGAT3-/- results in a frameshift mutation, and the E-PHA blot shows greatly reduced bisecting N-glycan, the study strongly supports that lowered GnT-III activity enhances cell invasion, growth, migration and cell-cell adhesion in NB. Our current and past studies have been summarized in an added figure, Fig 7, which clarifies results to strongly support our conclusion that similar phenotypic trends in NB cells are observed as GnT-III (MGAT3) activities are varied. We speculated that oligomannose and β1,6-GlcNAc branched N-glycans are key factors in altering cellular phenotypes. The speculation is based on observation of increased binding of GNL and L-PHA lectins which supported that oligomannose and β1,6-GlcNAc branched N-glycans were raised. Further, our past studies, along with several other studies which have related oligomannose to cell invasiveness, and β1,6-GlcNAc branched N-glycans to cell growth. Edits have been made in the abstract and throughout the manuscript to support the conclusion that reduced GnT-III activity promotes NB cellular properties which can easily be viewed in the marked version of the manuscript. Lack of funding to conduct Glycomic analysis on the various cell lines has limited our study and we hope to obtain funds in the future to conduct these studies.

2. Because MGAT3 deficiency affects multiple aspects of glycan processing simultaneously, the current data support association rather than direct causal separation of these glycan classes. These conclusions should therefore be moderated unless more direct evidence is provided.

**Authors:** The conclusions have been lessened to focus on reduced GnT-III activity (or lowered bisecting N-glycans) in the MGAT3-/- cell lines and increased GnT-III activity via transient expression, see our reply under comment 1.

3.It is not fully clear whether the BE(2)-C analysis is based on multiple independent knockout clones or essentially on a single selected clone. Since clonal variability can be substantial, this point should be stated clearly, and validation in an additional independent clone would strengthen the study.

**Authors:** We are using a single clone based on DNA sequencing and selection of cell colonies. This is stated in line 232-236 of results and described in materials and methods, lines: 105-119.

Of note, similar trends in phenotypes were observed in both knockout and overexpression of human BE(2)C cells and rat NB_1 cells, see heat map in Fig 7. Thus, human and rat share phenotypes due to altered GnT-III activity, along with our past studies of GnT-I and GnT-II (Hall et l., 2018; 2020; 2021;2023; 2024).

4. Transient re-expression of Mgat3 is useful, but overexpression may not simply restore the endogenous state. The limitations of this approach should be acknowledged more clearly.

**Authors:** We have reviewed and clearly stated that transient expression of cells were used to rescue the MGAT3-/- cell line, and for overexpression in the BE(2)-C and NB_1 cell lines. Clearly, we are aware that we did not completely restore the state of BE(2)-C, as not all cells are transfected and thus overexpressing the Mgat3 gene. However, when Mgat3 was overexpressed in the mutant line the cell properties tended towards the BE(2)-C cell line, not MGAT3-/-, and overexpression in the cell lines further reduced the cellular phenotypes, see Fig 7.

5.The manuscript should state more clearly what is treated as n in each assay. In several experiments, it appears that n may represent colonies, spheroids, or image fields rather than independent biological replicates. This distinction is important.

**Authors:** This has been reviewed and clarified as needed.

6. The current manuscript occasionally goes beyond what is directly demonstrated, particularly when assigning specific biological roles to distinct glycan classes or describing GnT-III as a suppressor of neuroblastoma progression. A more measured presentation would improve the manuscript.

**Authors:** Note, this has been addressed see our responses above.
