# Supplementary material for: Neuroblastoma cell growth and invasiveness is modulated by the activity of N-acetylglucosaminyltransferase-III
Source: PLoS One. 2026 Jun 8;21(6):e0350822. doi: 10.1371/journal.pone.0350822 (PMC13245742; doi:10.1371/journal.pone.0350822)
Supplement: Peer review history

# Peer review: Decision Letter 0

**DOI:** 10.1371/journal.pone.0350822.r001

**Authors:** Sushil Mishra

30 Mar 2026

PONE-D-25-68071

Neuroblastoma cell growth and invasiveness is modulated by the activity of N-acetylglucosaminyltransferase-III

PLOS One

Dear Dr. Schwalbe,

Thank you for submitting your manuscript to PLOS ONE. After careful consideration, we feel that it has merit but does not fully meet PLOS ONE’s publication criteria as it currently stands. Therefore, we invite you to submit a revised version of the manuscript that addresses the points raised during the review process.

Please submit your revised manuscript by May 14 2026 11:59PM. If you will need more time than this to complete your revisions, please reply to this message or contact the journal office at plosone@plos.org. When you're ready to submit your revision, log on to https://www.editorialmanager.com/pone/ and select the 'Submissions Needing Revision' folder to locate your manuscript file.

Please include the following items when submitting your revised manuscript:

- A letter that responds to each point raised by the academic editor and reviewer(s). You should upload this letter as a separate file labeled 'Response to Reviewers'.
- A marked-up copy of your manuscript that highlights changes made to the original version. You should upload this as a separate file labeled 'Revised Manuscript with Track Changes'.
- An unmarked version of your revised paper without tracked changes. You should upload this as a separate file labeled 'Manuscript'.

If you would like to make changes to your financial disclosure, please include your updated statement in your cover letter. Guidelines for resubmitting your figure files are available below the reviewer comments at the end of this letter.

If applicable, we recommend that you deposit your laboratory protocols in protocols.io to enhance the reproducibility of your results. Protocols.io assigns your protocol its own identifier (DOI) so that it can be cited independently in the future. For instructions see: https://journals.plos.org/plosone/s/submission-guidelines#loc-laboratory-protocols. Additionally, PLOS ONE offers an option for publishing peer-reviewed Lab Protocol articles, which describe protocols hosted on protocols.io. Read more information on sharing protocols at https://plos.org/protocols?utm_medium=editorial-email&utm_source=authorletters&utm_campaign=protocols.

As the corresponding author, your ORCID iD is verified in the submission system and will appear in the published article. PLOS supports the use of ORCID, and we encourage all coauthors to register for an ORCID iD and use it as well. Please encourage your coauthors to verify their ORCID iD within the submission system before final acceptance, as unverified ORCID iDs will not appear in the published article. *Only* the individual author can complete the verification step; PLOS staff *cannot* verify ORCID iDs on behalf of authors.

We look forward to receiving your revised manuscript.

Kind regards,

Sushil Mishra, Ph.D

Academic Editor

PLOS One

Journal Requirements:

When submitting your revision, we need you to address these additional requirements.

1.Please ensure that your manuscript meets PLOS ONE's style requirements, including those for file naming. The PLOS ONE style templates can be found at

https://journals.plos.org/plosone/s/file?id=wjVg/PLOSOne_formatting_sample_main_body.pdf and

https://journals.plos.org/plosone/s/file?id=ba62/PLOSOne_formatting_sample_title_authors_affiliations.pdf

2. We note that the grant information you provided in the ‘Funding Information’ and ‘Financial Disclosure’ sections do not match.

When you resubmit, please ensure that you provide the correct grant numbers for the awards you received for your study in the ‘Funding Information’ section.

3. Thank you for stating the following financial disclosure:

“This work was supported by the National Institutes of Health (NIH)-funded R15 grant (R15GM129679) to R.A.S.”

Please state what role the funders took in the study.  If the funders had no role, please state: "The funders had no role in study design, data collection and analysis, decision to publish, or preparation of the manuscript."

If this statement is not correct you must amend it as needed.

Please include this amended Role of Funder statement in your cover letter; we will change the online submission form on your behalf.

4. We note that your Data Availability Statement is currently as follows: All relevant data are within the manuscript and in Supporting Information files.

Please confirm at this time whether or not your submission contains all raw data required to replicate the results of your study. Authors must share the “minimal data set” for their submission. PLOS defines the minimal data set to consist of the data required to replicate all study findings reported in the article, as well as related metadata and methods (https://journals.plos.org/plosone/s/data-availability#loc-minimal-data-set-definition).

For example, authors should submit the following data:

- The values behind the means, standard deviations and other measures reported;

- The values used to build graphs;

- The points extracted from images for analysis.

Authors do not need to submit their entire data set if only a portion of the data was used in the reported study.

If your submission does not contain these data, please either upload them as Supporting Information files or deposit them to a stable, public repository and provide us with the relevant URLs, DOIs, or accession numbers. For a list of recommended repositories, please see https://journals.plos.org/plosone/s/recommended-repositories.

If there are ethical or legal restrictions on sharing a de-identified data set, please explain them in detail (e.g., data contain potentially sensitive information, data are owned by a third-party organization, etc.) and who has imposed them (e.g., an ethics committee). Please also provide contact information for a data access committee, ethics committee, or other institutional body to which data requests may be sent. If data are owned by a third party, please indicate how others may request data access.

5. Please include captions for your Supporting Information files at the end of your manuscript, and update any in-text citations to match accordingly. Please see our Supporting Information guidelines for more information: http://journals.plos.org/plosone/s/supporting-information.

6. PLOS ONE now requires that authors provide the original uncropped and unadjusted images underlying all blot or gel results reported in a submission’s figures or Supporting Information files. This policy and the journal’s other requirements for blot/gel reporting and figure preparation are described in detail at https://journals.plos.org/plosone/s/figures#loc-blot-and-gel-reporting-requirements and https://journals.plos.org/plosone/s/figures#loc-preparing-figures-from-image-files. When you submit your revised manuscript, please ensure that your figures adhere fully to these guidelines and provide the original underlying images for all blot or gel data reported in your submission. See the following link for instructions on providing the original image data: https://journals.plos.org/plosone/s/figures#loc-original-images-for-blots-and-gels.

In your cover letter, please note whether your blot/gel image data are in Supporting Information or posted at a public data repository, provide the repository URL if relevant, and provide specific details as to which raw blot/gel images, if any, are not available. Email us at plosone@plos.org if you have any questions.

7. If the reviewer comments include a recommendation to cite specific previously published works, please review and evaluate these publications to determine whether they are relevant and should be cited. There is no requirement to cite these works unless the editor has indicated otherwise.

[Note: HTML markup is below. Please do not edit.]

Reviewers' comments:

Reviewer's Responses to Questions

**Comments to the Author**

1. Is the manuscript technically sound, and do the data support the conclusions?

The manuscript must describe a technically sound piece of scientific research with data that supports the conclusions. Experiments must have been conducted rigorously, with appropriate controls, replication, and sample sizes. The conclusions must be drawn appropriately based on the data presented.

Reviewer #1: Yes

Reviewer #2: No

**********

2. Has the statistical analysis been performed appropriately and rigorously?

Reviewer #1: Yes

Reviewer #2: No

**********

3. Have the authors made all data underlying the findings in their manuscript fully available?

The PLOS Data policy requires authors to make all data underlying the findings described in their manuscript fully available without restriction, with rare exception (please refer to the Data Availability Statement in the manuscript PDF file). The data should be provided as part of the manuscript or its supporting information, or deposited to a public repository. For example, in addition to summary statistics, the data points behind means, medians and variance measures should be available. If there are restrictions on publicly sharing data—e.g. participant privacy or use of data from a third party—those must be specified.

Reviewer #1: Yes

Reviewer #2: Yes

**********

4. Is the manuscript presented in an intelligible fashion and written in standard English?

PLOS ONE does not copyedit accepted manuscripts, so the language in submitted articles must be clear, correct, and unambiguous. Any typographical or grammatical errors should be corrected at revision, so please note any specific errors here.

Reviewer #1: Yes

Reviewer #2: Yes

**********

5. Review Comments to the Author

Please use the space provided to explain your answers to the questions above. You may also include additional comments for the author, including concerns about dual publication, research ethics, or publication ethics. (Please upload your review as an attachment if it exceeds 20,000 characters)

Reviewer #1: In this manuscript, Burch et al. reports the study on the influential role of GnT-III on NB growth, cell-cell adhesion, invasion, and migration. GnT-III has long been considered as a tumor suppressor. This study created human MGAT3 knockout NB cells, and found the functional loss of GnT-III caused a decline of bisected N-glycans, and increase of β1,6-GlcNAc branched and oligomannose type N-glycans. The results suggest the importance of GnT-III activity in NB cell growth and invasiveness. The experimental details and characterization are well-documented, and the subject area and findings are of considerable importance. It is sound for publication in the PLOS One, however, a few points should be addressed before acceptance.

In text and figures, the refer of cell lines, including the wt and knockout cells are somehow not clear. It is suggested to clarify the description (BE2-C or NB-1, wt or knockout or GnT-III complementary).

A shematic diagram of the correlation among bisected N-glycans, β1,6-GlcNAc branched and oligomannose type N-glycans could be provided to benefit the understanding of this work.

Reviewer #2: This manuscript examines the effects of MGAT3/GnT-III deficiency on neuroblastoma cell behavior using a human BE(2)-C MGAT3 knockout model together with complementary experiments in a rat NB_1 system. The topic is interesting, and the phenotypic dataset is relatively broad, including proliferation, anchorage-independent growth, migration, spheroid invasion, cell-cell adhesion, and morphology. Overall, the study has merit and may become suitable for publication after major revision. However, in its current form, several mechanistic conclusions are stronger than the data support.

1. At present, the conclusions rely heavily on lectin blotting, which is useful but does not allow a sufficiently precise definition of the underlying structural changes. In particular, the current data do not clearly distinguish whether the observed phenotypes are attributable specifically to the loss of bisecting GlcNAc itself, to altered β1,6 branching, to changes in sialylation, or to broader remodeling of other glycan structures. More generally, it also remains unclear whether the phenotypes should be interpreted specifically in terms of N-glycan alterations alone. Because MGAT3 deficiency would be expected to affect glycan processing more broadly, the authors should avoid assigning the observed phenotypes too directly to a single glycan feature unless additional structural evidence is provided. Ideally, this point should be addressed by more detailed glycomics analyses. Alternatively, a broader multi-panel lectin blot analysis would at least help to define the glycan changes more comprehensively.

2. Because MGAT3 deficiency affects multiple aspects of glycan processing simultaneously, the current data support association rather than direct causal separation of these glycan classes. These conclusions should therefore be moderated unless more direct evidence is provided.

3.It is not fully clear whether the BE(2)-C analysis is based on multiple independent knockout clones or essentially on a single selected clone. Since clonal variability can be substantial, this point should be stated clearly, and validation in an additional independent clone would strengthen the study.

4. Transient re-expression of Mgat3 is useful, but overexpression may not simply restore the endogenous state. The limitations of this approach should be acknowledged more clearly.

5.The manuscript should state more clearly what is treated as n in each assay. In several experiments, it appears that n may represent colonies, spheroids, or image fields rather than independent biological replicates. This distinction is important.

6. The current manuscript occasionally goes beyond what is directly demonstrated, particularly when assigning specific biological roles to distinct glycan classes or describing GnT-III as a suppressor of neuroblastoma progression. A more measured presentation would improve the manuscript.

**********

6. PLOS authors have the option to publish the peer review history of their article (what does this mean?). If published, this will include your full peer review and any attached files.

If you choose “no”, your identity will remain anonymous but your review may still be made public.

**Do you want your identity to be public for this peer review?** For information about this choice, including consent withdrawal, please see our Privacy Policy.

Reviewer #1: No

Reviewer #2: No

**********

[NOTE: If reviewer comments were submitted as an attachment file, they will be attached to this email and accessible via the submission site. Please log into your account, locate the manuscript record, and check for the action link "View Attachments". If this link does not appear, there are no attachment files.]

To ensure your figures meet our technical requirements, please review our figure guidelines: https://journals.plos.org/plosone/s/figures

You may also use PLOS’s free figure tool, NAAS, to help you prepare publication quality figures: https://journals.plos.org/plosone/s/figures#loc-tools-for-figure-preparation.

NAAS will assess whether your figures meet our technical requirements by comparing each figure against our figure specifications.

# Author comment: Author response to Decision Letter 1

**DOI:** 10.1371/journal.pone.0350822.r002

13 Apr 2026

POINT-BY-PONT RESPONSE TO REVIEWERS’

Reviewer #1: In this manuscript, Burch et al. reports the study on the influential role of GnT-III on NB growth, cell-cell adhesion, invasion, and migration. GnT-III has long been considered as a tumor suppressor. This study created human MGAT3 knockout NB cells, and found the functional loss of GnT-III caused a decline of bisected N-glycans, and increase of β1,6-GlcNAc branched and oligomannose type N-glycans. The results suggest the importance of GnT-III activity in NB cell growth and invasiveness. The experimental details and characterization are well-documented, and the subject area and findings are of considerable importance. It is sound for publication in the PLOS One, however, a few points should be addressed before acceptance.

In text and figures, the refer of cell lines, including the wt and knockout cells are somehow not clear. It is suggested to clarify the description (BE2-C or NB-1, wt or knockout or GnT-III complementary).

Authors: We have changed Wt to Be(2)-C and checked the wording throughout to clarify names of cell lines. Since Be(2)-C is a human cell line the mutant cell line is noted as MGAT3-/- while the NB_1 cell is a rat NB cell lines the mutant is referred to as Mgat3-/-.

A shematic diagram of the correlation among bisected N-glycans, β1,6-GlcNAc branched and oligomannose type N-glycans could be provided to benefit the understanding of this work.

Authors: Since Glycomic analysis was not completed, we have concluded the reduced GnT-III activity (or lowered bisecting N-glycans) increased cell invasiveness, growth, migration, and cell-cell adhesion. The results from this study and our past study strongly support our conclusion that similar phenotypic trends in NB cells are observed as GnT-III (MGAT3) activities are varied, see our added figure, Fig 7. We have speculated the involvement of oligomannose and β1,6-GlcNAc branched N-glycans in altering these cellular phenotypes. See edits in abstract and throughout text of manuscript which can easily be viewed in marker version of the manuscript. Our future goal is to obtain funding to conduct Glycomic analysis.

Reviewer #2: This manuscript examines the effects of MGAT3/GnT-III deficiency on neuroblastoma cell behavior using a human BE(2)-C MGAT3 knockout model together with complementary experiments in a rat NB_1 system. The topic is interesting, and the phenotypic dataset is relatively broad, including proliferation, anchorage-independent growth, migration, spheroid invasion, cell-cell adhesion, and morphology. Overall, the study has merit and may become suitable for publication after major revision. However, in its current form, several mechanistic conclusions are stronger than the data support.

1. At present, the conclusions rely heavily on lectin blotting, which is useful but does not allow a sufficiently precise definition of the underlying structural changes. In particular, the current data do not clearly distinguish whether the observed phenotypes are attributable specifically to the loss of bisecting GlcNAc itself, to altered β1,6 branching, to changes in sialylation, or to broader remodeling of other glycan structures. More generally, it also remains unclear whether the phenotypes should be interpreted specifically in terms of N-glycan alterations alone. Because MGAT3 deficiency would be expected to affect glycan processing more broadly, the authors should avoid assigning the observed phenotypes too directly to a single glycan feature unless additional structural evidence is provided. Ideally, this point should be addressed by more detailed glycomics analyses. Alternatively, a broader multi-panel lectin blot analysis would at least help to define the glycan changes more comprehensively.

Authors: This study related the phenotypes to various types of glycans, not a single glycan structure. We do agree that glycomic analysis is needed to strongly support that oligomannose and β1,6-GlcNAc branched N-glycans are raised in the MGAT3-/- cell line and thus contributing to cell invasiveness and growth, respectively. We also wish to add the following for clarification purposes. Since the genetic modification in MGAT3-/- results in a frameshift mutation, and the E-PHA blot shows greatly reduced bisecting N-glycan, the study strongly supports that lowered GnT-III activity enhances cell invasion, growth, migration and cell-cell adhesion in NB. Our current and past studies have been summarized in an added figure, Fig 7, which clarifies results to strongly support our conclusion that similar phenotypic trends in NB cells are observed as GnT-III (MGAT3) activities are varied. We speculated that oligomannose and β1,6-GlcNAc branched N-glycans are key factors in altering cellular phenotypes. The speculation is based on observation of increased binding of GNL and L-PHA lectins which supported that oligomannose and β1,6-GlcNAc branched N-glycans were raised. Further, our past studies, along with several other studies which have related oligomannose to cell invasiveness, and β1,6-GlcNAc branched N-glycans to cell growth. Edits have been made in the abstract and throughout the manuscript to support the conclusion that reduced GnT-III activity promotes NB cellular properties which can easily be viewed in the marked version of the manuscript. Lack of funding to conduct Glycomic analysis on the various cell lines has limited our study and we hope to obtain funds in the future to conduct these studies.

2. Because MGAT3 deficiency affects multiple aspects of glycan processing simultaneously, the current data support association rather than direct causal separation of these glycan classes. These conclusions should therefore be moderated unless more direct evidence is provided.

Authors: The conclusions have been lessened to focus on reduced GnT-III activity (or lowered bisecting N-glycans) in the MGAT3-/- cell lines and increased GnT-III activity via transient expression, see our reply under comment 1.

3.It is not fully clear whether the BE(2)-C analysis is based on multiple independent knockout clones or essentially on a single selected clone. Since clonal variability can be substantial, this point should be stated clearly, and validation in an additional independent clone would strengthen the study.

Authors: We are using a single clone based on DNA sequencing and selection of cell colonies. This is stated in line 232-236 of results and described in materials and methods, lines: 105-119.

Of note, similar trends in phenotypes were observed in both knockout and overexpression of human BE(2)C cells and rat NB_1 cells, see heat map in Fig 7. Thus, human and rat share phenotypes due to altered GnT-III activity, along with our past studies of GnT-I and GnT-II (Hall et l., 2018; 2020; 2021;2023; 2024).

4. Transient re-expression of Mgat3 is useful, but overexpression may not simply restore the endogenous state. The limitations of this approach should be acknowledged more clearly.

Authors: We have reviewed and clearly stated that transient expression of cells were used to rescue the MGAT3-/- cell line, and for overexpression in the BE(2)-C and NB_1 cell lines. Clearly, we are aware that we did not completely restore the state of BE(2)-C, as not all cells are transfected and thus overexpressing the Mgat3 gene. However, when Mgat3 was overexpressed in the mutant line the cell properties tended towards the BE(2)-C cell line, not MGAT3-/-, and overexpression in the cell lines further reduced the cellular phenotypes, see Fig 7.

5.The manuscript should state more clearly what is treated as n in each assay. In several experiments, it appears that n may represent colonies, spheroids, or image fields rather than independent biological replicates. This distinction is important.

Authors: This has been reviewed and clarified as needed.

6. The current manuscript occasionally goes beyond what is directly demonstrated, particularly when assigning specific biological roles to distinct glycan classes or describing GnT-III as a suppressor of neuroblastoma progression. A more measured presentation would improve the manuscript.

Authors: Note, this has been addressed see our responses above.

Attachment Submitted filename: 260408_RESPONSE TO REVIEWERS.docx

# Peer review: Decision Letter 1

**DOI:** 10.1371/journal.pone.0350822.r003

**Authors:** Sushil Mishra

8 May 2026

PONE-D-25-68071R1

Neuroblastoma cell growth and invasiveness is modulated by the activity of N-acetylglucosaminyltransferase-III

PLOS One

Dear Dr. Schwalbe,

Thank you for submitting your manuscript to PLOS ONE. After careful consideration, we feel that it has merit but does not fully meet PLOS ONE’s publication criteria as it currently stands. Therefore, we invite you to submit a revised version of the manuscript that addresses the points raised during the review process.

Please submit your revised manuscript by Jun 22 2026 11:59PM. If you will need more time than this to complete your revisions, please reply to this message or contact the journal office at plosone@plos.org. When you're ready to submit your revision, log on to https://www.editorialmanager.com/pone/ and select the 'Submissions Needing Revision' folder to locate your manuscript file.

Please include the following items when submitting your revised manuscript:

- A letter that responds to each point raised by the academic editor and reviewer(s). You should upload this letter as a separate file labeled 'Response to Reviewers'.
- A marked-up copy of your manuscript that highlights changes made to the original version. You should upload this as a separate file labeled 'Revised Manuscript with Track Changes'.
- An unmarked version of your revised paper without tracked changes. You should upload this as a separate file labeled 'Manuscript'.

If you would like to make changes to your financial disclosure, please include your updated statement in your cover letter. Guidelines for resubmitting your figure files are available below the reviewer comments at the end of this letter.

If applicable, we recommend that you deposit your laboratory protocols in protocols.io to enhance the reproducibility of your results. Protocols.io assigns your protocol its own identifier (DOI) so that it can be cited independently in the future. For instructions see: https://journals.plos.org/plosone/s/submission-guidelines#loc-laboratory-protocols. Additionally, PLOS ONE offers an option for publishing peer-reviewed Lab Protocol articles, which describe protocols hosted on protocols.io. Read more information on sharing protocols at https://plos.org/protocols?utm_medium=editorial-email&utm_source=authorletters&utm_campaign=protocols.

As the corresponding author, your ORCID iD is verified in the submission system and will appear in the published article. PLOS supports the use of ORCID, and we encourage all coauthors to register for an ORCID iD and use it as well. Please encourage your coauthors to verify their ORCID iD within the submission system before final acceptance, as unverified ORCID iDs will not appear in the published article. *Only* the individual author can complete the verification step; PLOS staff *cannot* verify ORCID iDs on behalf of authors.

We look forward to receiving your revised manuscript.

Kind regards,

Sushil Mishra, Ph.D

Academic Editor

PLOS One

**Journal Requirements:**

1. If the reviewer comments include a recommendation to cite specific previously published works, please review and evaluate these publications to determine whether they are relevant and should be cited. There is no requirement to cite these works unless the editor has indicated otherwise.

2. Please review your reference list to ensure that it is complete and correct. If you have cited papers that have been retracted, please include the rationale for doing so in the manuscript text, or remove these references and replace them with relevant current references. Any changes to the reference list should be mentioned in the rebuttal letter that accompanies your revised manuscript. If you need to cite a retracted article, indicate the article’s retracted status in the References list and also include a citation and full reference for the retraction notice.

[Note: HTML markup is below. Please do not edit.]

Reviewers' comments:

Reviewer's Responses to Questions

**Comments to the Author**

1. If the authors have adequately addressed your comments raised in a previous round of review and you feel that this manuscript is now acceptable for publication, you may indicate that here to bypass the “Comments to the Author” section, enter your conflict of interest statement in the “Confidential to Editor” section, and submit your "Accept" recommendation.

Reviewer #1: (No Response)

Reviewer #2: (No Response)

**********

2. Is the manuscript technically sound, and do the data support the conclusions?

The manuscript must describe a technically sound piece of scientific research with data that supports the conclusions. Experiments must have been conducted rigorously, with appropriate controls, replication, and sample sizes. The conclusions must be drawn appropriately based on the data presented.

Reviewer #1: Yes

Reviewer #2: Yes

**********

3. Has the statistical analysis been performed appropriately and rigorously?

Reviewer #1: Yes

Reviewer #2: Yes

**********

4. Have the authors made all data underlying the findings in their manuscript fully available?

The PLOS Data policy requires authors to make all data underlying the findings described in their manuscript fully available without restriction, with rare exception (please refer to the Data Availability Statement in the manuscript PDF file). The data should be provided as part of the manuscript or its supporting information, or deposited to a public repository. For example, in addition to summary statistics, the data points behind means, medians and variance measures should be available. If there are restrictions on publicly sharing data—e.g. participant privacy or use of data from a third party—those must be specified.

Reviewer #1: Yes

Reviewer #2: Yes

**********

5. Is the manuscript presented in an intelligible fashion and written in standard English?

PLOS ONE does not copyedit accepted manuscripts, so the language in submitted articles must be clear, correct, and unambiguous. Any typographical or grammatical errors should be corrected at revision, so please note any specific errors here.

Reviewer #1: Yes

Reviewer #2: Yes

**********

6. Review Comments to the Author

Please use the space provided to explain your answers to the questions above. You may also include additional comments for the author, including concerns about dual publication, research ethics, or publication ethics. (Please upload your review as an attachment if it exceeds 20,000 characters)

**Reviewer #1:** After addressing the following two points, this manuscript could be accepted for publication in the PLOS One.

Line 45, please optimize the descripiton “modifying N-glycan expression”.

Please unify the descripition of human Be(2)-C NB and Be(2)-C cell lines in the manuscript.

**Reviewer #2:** Overall, the authors have addressed most of the concerns raised. However, it would further strengthen the manuscript if the limitations of the study were more explicitly acknowledged in the Discussion. In particular, the absence of comprehensive glycomics analysis and the potential impact of clonal variability (given that the knockout analysis is based on a single clone) should be clearly stated as limitations of this work. Explicitly addressing these points would help to better contextualize the conclusions and improve the overall rigor of the study.

**********

7. PLOS authors have the option to publish the peer review history of their article (what does this mean?). If published, this will include your full peer review and any attached files.

If you choose “no”, your identity will remain anonymous but your review may still be made public.

**Do you want your identity to be public for this peer review?** For information about this choice, including consent withdrawal, please see our Privacy Policy.

Reviewer #1: No

Reviewer #2: **Yes:** Hirokazu Yagi

**********

[NOTE: If reviewer comments were submitted as an attachment file, they will be attached to this email and accessible via the submission site. Please log into your account, locate the manuscript record, and check for the action link "View Attachments". If this link does not appear, there are no attachment files.]

To ensure your figures meet our technical requirements, please review our figure guidelines: https://journals.plos.org/plosone/s/figures

You may also use PLOS’s free figure tool, NAAS, to help you prepare publication quality figures: https://journals.plos.org/plosone/s/figures#loc-tools-for-figure-preparation.

NAAS will assess whether your figures meet our technical requirements by comparing each figure against our figure specifications.

# Author comment: Author response to Decision Letter 2

**DOI:** 10.1371/journal.pone.0350822.r004

12 May 2026

POINT-BY-POINT RESPONSE TO REVIEWERS

Reviewer #1: After addressing the following two points, this manuscript could be accepted for publication in PLOS ONE.

Line 45: please revise the wording around “modifying N-glycan expression” for clarity.

Authors’ response: We revised this sentence to specify the relevant genes and clarify that changes in glycosyltransferase expression alter N-glycan branching (number and elongation of branches).

Please use consistent terminology when referring to the human BE(2)-C neuroblastoma (NB) and BE(2)-C cell lines throughout the manuscript.

Authors response: This has been addressed throughout the manuscript. We have used the BE(2)-C cell line.

Reviewer #2: Overall, the authors have addressed most of the concerns raised. However, the manuscript would be strengthened by a more explicit discussion of study limitations. In particular, please note (1) the lack of comprehensive glycomics analysis and (2) the potential impact of clonal variability, given that the knockout analysis is based on a single clone. Clearly stating these limitations will better contextualize the conclusions and strengthen the rigor of the study.

Authors response: This concern has been addressed, see last paragraph in the discussion section.

Attachment Submitted filename: Response to reviewers.pdf

# Editor report: Decision Letter 2

**DOI:** 10.1371/journal.pone.0350822.r005

**Authors:** Sushil Mishra

20 May 2026

Neuroblastoma cell growth and invasiveness is modulated by the activity of N-acetylglucosaminyltransferase-III

PONE-D-25-68071R2

Dear Dr. Schwalbe,

We’re pleased to inform you that your manuscript has been judged scientifically suitable for publication and will be formally accepted for publication once it meets all outstanding technical requirements.

Within one week, you’ll receive an e-mail detailing the required amendments. When these have been addressed, you’ll receive a formal acceptance letter and your manuscript will be scheduled for publication.

An invoice will be generated when your article is formally accepted. Please note, if your institution has a publishing partnership with PLOS and your article meets the relevant criteria, all or part of your publication costs will be covered. Please make sure your user information is up-to-date by logging into Editorial Manager at Editorial Manager® and clicking the ‘Update My Information' link at the top of the page. For questions related to billing, please contact billing support.

If your institution or institutions have a press office, please notify them about your upcoming paper to help maximize its impact. If they’ll be preparing press materials, please inform our press team as soon as possible -- no later than 48 hours after receiving the formal acceptance. Your manuscript will remain under strict press embargo until 2 pm Eastern Time on the date of publication. For more information, please contact onepress@plos.org.

Kind regards,

Sushil Mishra, Ph.D

Academic Editor

PLOS One

# Editor report: Acceptance letter

**DOI:** 10.1371/journal.pone.0350822.r006

**Authors:** Sushil Mishra

PONE-D-25-68071R2

PLOS One

Dear Dr. Schwalbe,

I'm pleased to inform you that your manuscript has been deemed suitable for publication in PLOS One. Congratulations! Your manuscript is now being handed over to our production team.

At this stage, our production department will prepare your paper for publication. This includes ensuring the following:

* All references, tables, and figures are properly cited

* All relevant supporting information is included in the manuscript submission,

* There are no issues that prevent the paper from being properly typeset

You will receive further instructions from the production team, including instructions on how to review your proof when it is ready. Please keep in mind that we are working through a large volume of accepted articles, so please give us a few days to review your paper and let you know the next and final steps.

Lastly, if your institution or institutions have a press office, please let them know about your upcoming paper now to help maximize its impact. If they'll be preparing press materials, please inform our press team within the next 48 hours. Your manuscript will remain under strict press embargo until 2 pm Eastern Time on the date of publication. For more information, please contact onepress@plos.org.

You will receive an invoice from PLOS for your publication fee after your manuscript has reached the completed accept phase. If you receive an email requesting payment before acceptance or for any other service, this may be a phishing scheme. Learn how to identify phishing emails and protect your accounts at https://explore.plos.org/phishing.

If we can help with anything else, please email us at customercare@plos.org.

Thank you for submitting your work to PLOS ONE and supporting open access.

Kind regards,

PLOS ONE Editorial Office Staff

on behalf of

Dr. Sushil Mishra

Academic Editor

PLOS One
